# Supplementary material for: Water Softening Using a Light-Responsive, Spiropyran-Modified Nanofiltration Membrane
Source: Polymers (Basel). 2019 Feb 15;11(2):344. doi: 10.3390/polym11020344 (PMC6419182; doi:10.3390/polym11020344)
Supplement: Supplementary file 1 [file polymers-11-00344-s001.pdf]

Supporting Information

Water Softening Using a Light-Responsive, Spiropyran-Modified Nanofiltration Membrane

Rasel Das,[1] Mathias Kuehnert,[1] Asieh Sadatkazemi,[2] Yaser Abdi,[2] and Agnes Schulze[1,*]

[1] Leibniz Institute of Surface Engineering (IOM), Permoserstr. 15, 04318 Leipzig, Germany

[2] Nanophysics Research Laboratory, Department of Physics, University of Tehran

***** Correspondence: agnes.schulze@iom-leipzig.de; Tel.: +49-341-235-2400

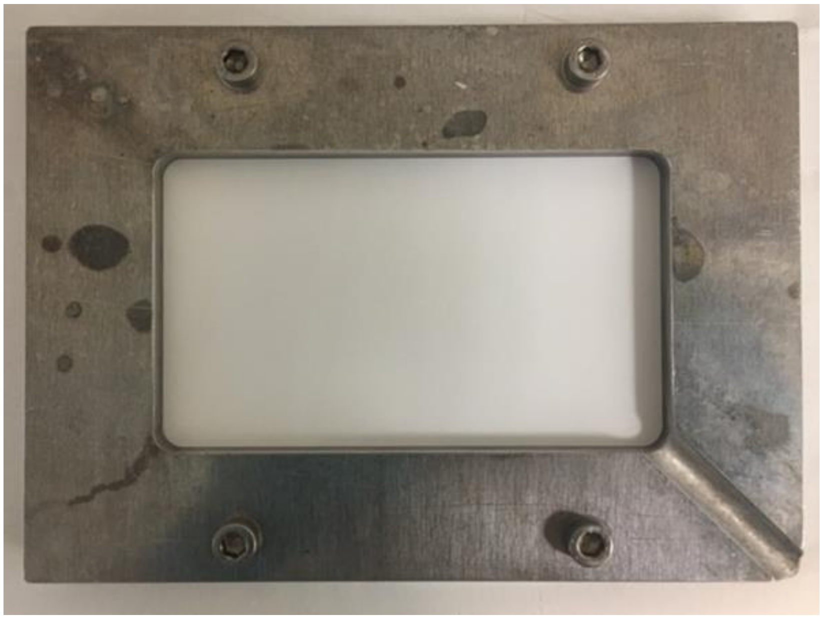

Figure S1. Clamping of PES using two stainless steel frames for controlling the interfacial polymerization on the top side of the PES support membrane.

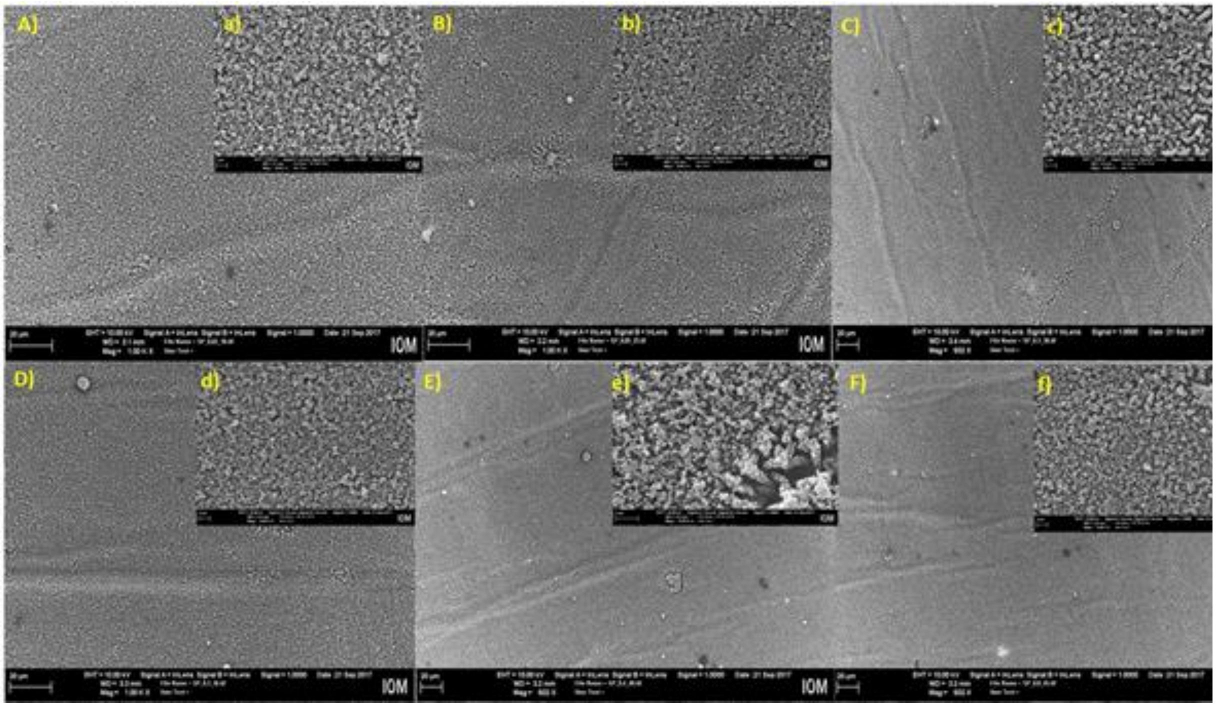

Figure S2. Low-resolution SEM images of PES/PA-TFC-SP membranes treated with 0.02 (**A**), 0.05 (**B**), 0.10 (**C**), 0.20 (**D**), 0.40 (**E**), and 0.80 wt % SP (**F**) with their corresponding high-resolution images (inset).

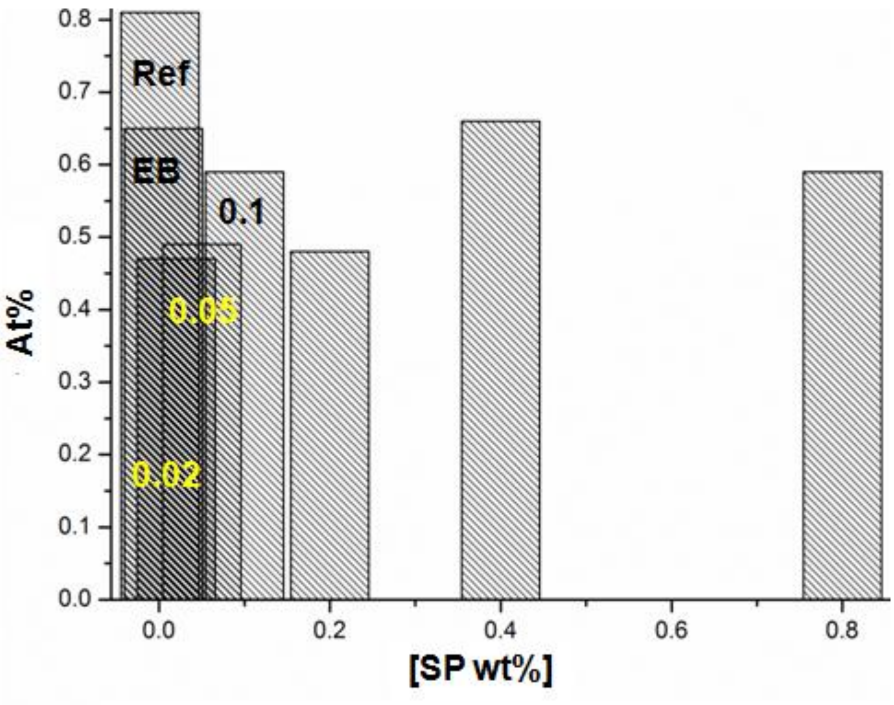

Figure S3. XPS atomic percentage of chlorine in reference PES/PA-TFC, EB-treated, and PES/PA-TFC-SP membranes.
